# Sclerotic chronic cutaneous graft-versus-host-disease following pseudoallogeneic chimeric antigen receptor T-cell therapy

**DOI:** 10.1016/j.jdcr.2024.06.014

**Published:** 2024-07-06

**Authors:** Esther Choi, Cuong V. Nguyen

**Affiliations:** a Department of Dermatology, Feinberg School of Medicine, Northwestern University, Chicago, Illinois; b Washington State University Elson S. Floyd College of Medicine, Spokane, Washington

**Keywords:** allogeneic-hematologic stem cell transplant, chimeric antigen receptor T-cell, donor lymphocyte infusion, graft-versus-host disease, GVHD, ScGVHD, sclerotic cutaneous graft-versus-host disease

## Introduction

Chronic cutaneous graft-versus-host disease (GVHD) represents a common complication following allogeneic-hematologic stem cell transplant (allo-HSCT). Although GVHD has been well characterized in the context of allo-HSCT, autologous chimeric antigen receptor T-cell (CAR-T) therapy should, in theory, not exhibit self-reactivity because of thymic selection. However, it is important to note that patients may undergo CAR-T therapy after relapsing from allo-HSCT. Thus, the engineering of T cells procured from an allo-HSCT recipient with >95% donor chimerism is termed pseudoallogenic CAR-T therapy, given the likelihood of the engineered T cells originating from donor cell lines.^1^ Herein, we present a unique case of a patient who was diagnosed with sclerotic chronic GVHD (ScGVHD) 15 months following CAR-T therapy.

## Case report

**Fig 1:**
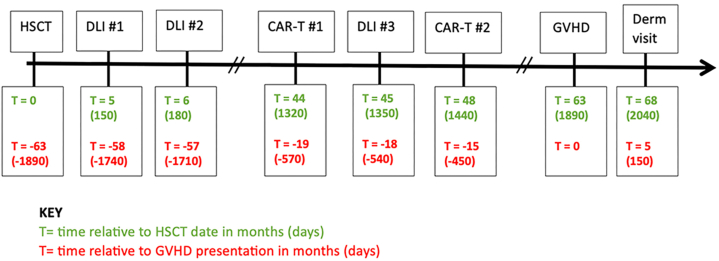
Timeline of sclerotic chronic GVHD relative to time from allogeneic-hematologic stem cell transplant and time from chimeric antigen receptor T-cell therapy. *CAR-T*, Chimeric antigen receptor T-cell therapy; *Derm*, dermatology; *DLI*, donor lymphocyte infusion; *GVHD*, graft-versus-host disease; *HSCT*, hematologic stem cell transplant.

A 56-year-old woman presented with skin dyspigmentation, generalized erythema, and focal thickening of the upper portion of the back and upper portion of the arms. Her past medical history was significant for chronic lymphocytic leukemia with Richter transformation to diffuse large B-cell lymphoma. In addition to various chemotherapies, her treatment course included allo-HSCT with a matched sibling donor in 2016 followed by 2 donor lymphocyte infusions (DLIs) (dose 1 × 10^7^ CD3^+^/kg) within 6 months of allo-HSCT for persistent disease. She experienced a recurrence of the disease and was treated with autologous CAR-T therapy with fludarabine and cyclophosphamide for lymphodepletion 44 months after HSCT. This was complicated by persistent transfusion-dependent pancytopenia, requiring DLI 1 month after CAR-T (dose 8 × 10^6^ CD34^+^/kg), collected from her original HSCT donor. Restaging scans demonstrated progression, and the decision was made to repeat auto-CAR-T with dose reduced fludarabine and cyclophosphamide conditioning 4 months after her first CAR-T therapy. Her course was further complicated by immune-mediated myelotoxcity from CAR-T and hypogammaglobinemia requiring monthly intravenous immunoglobulin infusions. Until the current presentation, the patient had not exhibited cutaneous or systemic GVHD and she had not taken immunosuppression for GVHD prophylaxis since her allo-HSCT. See Fig 1 for the full timeline of the patient’s clinical presentation.

**Fig 2:**
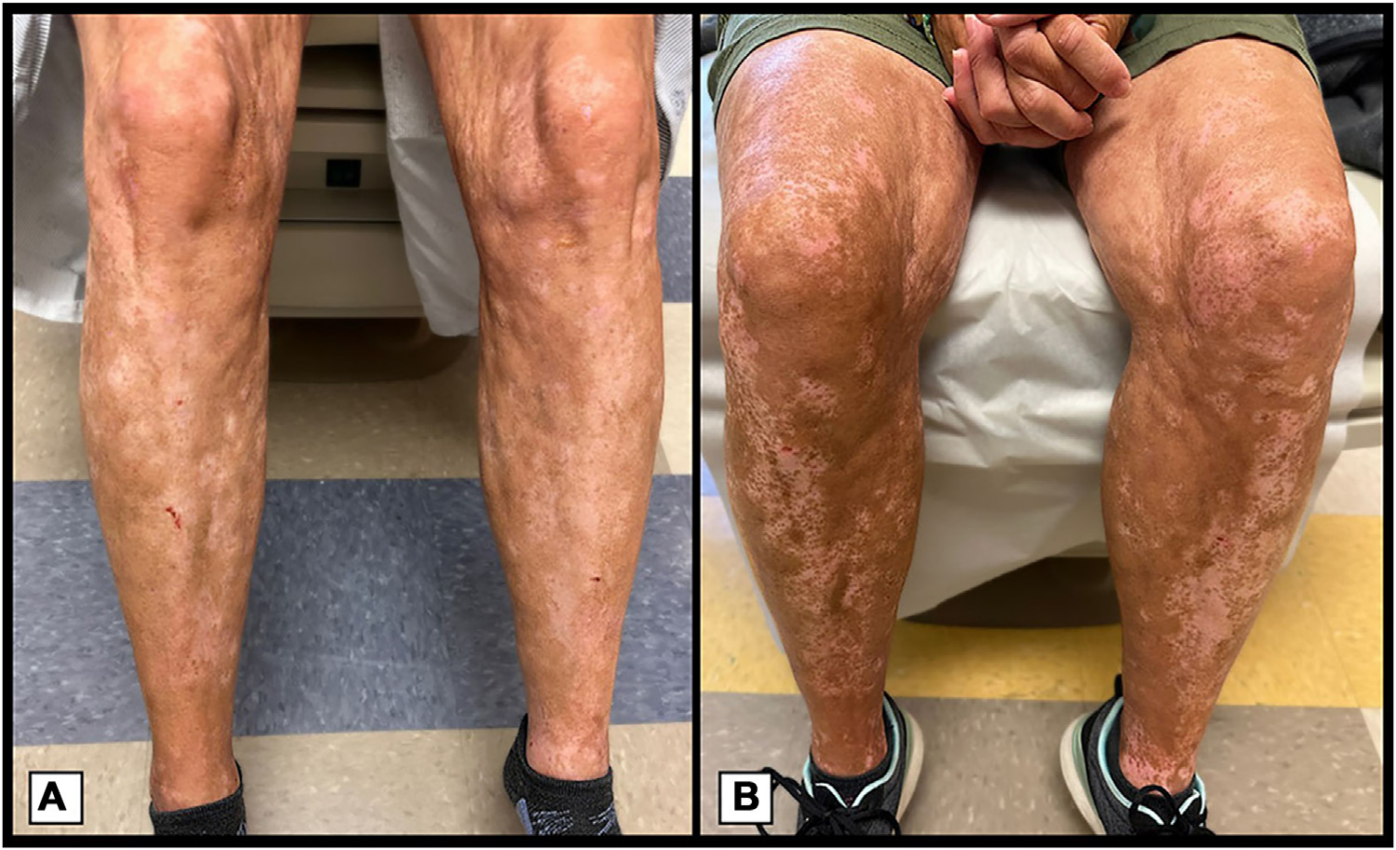
**A** and **B,** Progression of sclerotic chronic graft-versus-host disease with “salt-and-pepper” dyschromia on the lower extremities within 6 months.

**Fig 3:**
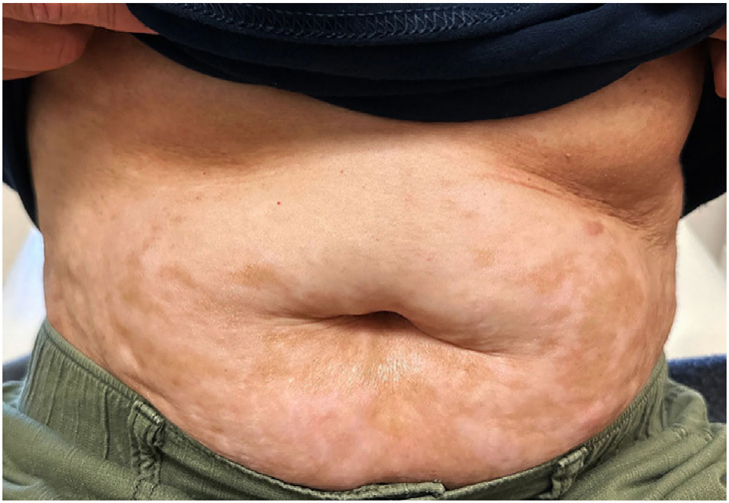
Hypopigmented patches on the base of sclerotic skin, forming rippling, and linear grooves on the abdomen.

**Fig 4:**
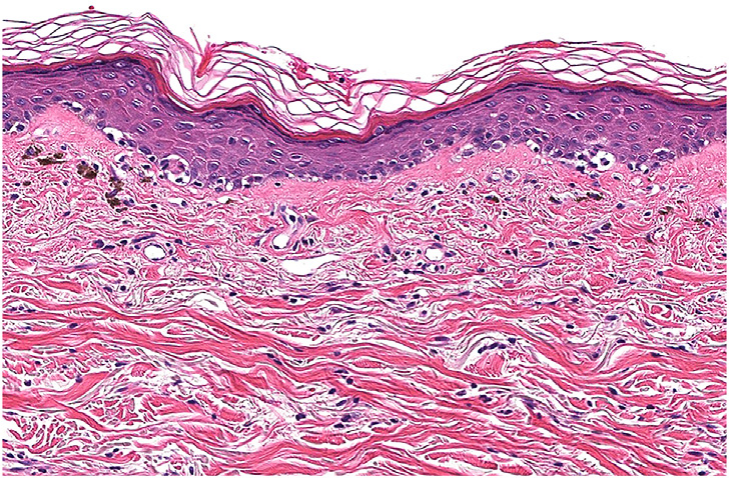
Vacuolar interface dermatitis with lymphocytes abutting the dermoepidermal junction and scattered necrotic keratinocytes consistent with grade II cutaneous graft-versus-host disease. (Hematoxylin-eosin stain; original magnification: ×130.)

At the time of our initial evaluation, her leukemia was in remission, and she had achieved 100% donor chimerism for 2 years. Laboratory investigations revealed an elevated antinuclear antibody titer at 1:1280 but a negative reflexive panel. Subsequent visits demonstrated further sclerosis, forming curvy linear grooves on the trunk, upper and lower extremities, and progressive “salt-and-pepper” dyschromia, consistent with ScGVHD (Figs 2 and 3), as well as GVHD involving her joints causing stiffness. Skin biopsy from her initial visit exhibited a mild interface reaction with periadnexal involvement consistent with cutaneous GVHD (Fig 4). Treatments included topical steroids, ruxolitinib 10 to 20 mg, and 5 infusions of rituximab with subsequent moderate softening of her skin, but slow repigmentation.

## Discussion

The safety and efficacy of pseudoallogeneic CAR-T therapy have been promising. However, the risk of GVHD development following therapy remains uncertain. This data paucity may be due, in part, to the limited sample sizes of CAR-T therapy studies, often with <50 patients in total. The incidence of acute and chronic GVHD of any organ type in this patient population has ranged from 0% to 50% with sparse information on skin disease presentation.^1^, ^2^, ^3^ Changes of ScGVHD, as in this case, have not been previously reported.

ScGVHD can mimic other sclerosing disorders, such as systemic sclerosis, morphea, and eosinophilic fasciitis, as well as macular hyperpigmentation disorders such as lichen planus pigmentosus or erythema dyschromicum perstans. Although all these conditions were considered, the lack of classic autoimmune connective disease features of Raynaud phenomenon, digital ulcerations, flexion contractures, coupled with the patient’s biopsy findings, ScGVHD was favored.

As most of these studies exclude patients with pre-existing GVHD, the effect of pseudoallogeneic CAR-T therapy on prior GVHD is also undefined. Interestingly, one report described a reversal of GVHD symptoms post-CAR-T therapy.^4^ The risk of GVHD development may be higher in settings where CAR-T is delivered within 10 months following allo-HSCT, particularly with the use of second-generation CAR-T.^1^^,^^3^ In line with prior studies, our patient received a second-generation CD19-directed CAR-T with a 4-1BB costimulatory domain, albeit this therapy was delivered 44 months after allo-HSCT.

In addition, the onset of ScGVHD occurs at a mean of 529 days after HSCT (range, 193-1001 days).^5^ First signs of skin changes developed in our patient approximately 450 days after the last CAR-T treatment and approximately 1900 days after her allo-HSCT. Her cutaneous symptoms may be more suggestive of a CAR-T complication, but the impact of DLIs following CAR-T therapy may have also played a clinical role in GVHD occurrence.

DLI is given with the intent of eliciting graft-versus-tumor effect after relapse from allo-HSCT. Unlike CAR-T where T cells are engineered to target malignant cells, the effects of DLI are less selective and have been associated with the induction of GVHD in 30% to 60% of patients.^6^ The onset of chronic GVHD after the initial infusion of DLI typically occurs at a median of 135 days (IQR, 89-237).^7^ Risk factors for GVHD after DLI include >60 years old, interval from HSCT to first infusion of DLI <184 days, and history of GVHD post-HSCT but pre-DLI.^7^ Our patient had no symptoms of cutaneous or systemic GVHD before her current ScGVHD development. Although our patient received her first DLI infusion approximately 150 days after HSCT, she exhibited first signs of cutaneous GVHD far beyond the typical IQR following DLI. Her ScGVHD symptoms developed just over 1740 days after her initial DLI infusion and approximately 450 days after her last DLI infusion. To our knowledge, only 1 other report has detailed the development of GVHD in patients who underwent CAR-T therapy following HSCT with or without DLI; of the 7 patients included, 2 developed new onset of GVHD post-CAR-T therapy, whereas none of these patients had a history of DLI. In contrast, 2 patients who received DLI following CAR-T therapy did not experience GVHD.^8^

As exemplified here, data concerning the incidence of GVHD and the role that CAR-T plays in its development require longer-term follow-up. GVHD is a complex, multifactorial process and the natural history and evolution of ScGVHD have yet to be elucidated. Although discerning the etiology of GVHD in patients with extensive pretreatment histories, such as ours, is challenging, we believe it is important to share the potential of this paradoxical phenomenon in patients with CAR-T therapy, given the anticipated expansion and utilization of this treatment modality against hematologic and solid organ tumors. We highlight the need for vigilant monitoring of patients undergoing CAR-T for the potential development of GVHD and other autoreactive cutaneous complications.

## Conflicts of interest

None disclosed.

## References

[1] Siglin J., Lutfi F., Bukhari A. (2020). Pseudo-allogeneic CAR-T therapy after allogeneic stem cell transplantation in relapsed/refractory B-cell NHL. Blood.

[2] Gardner R.A., Finney O., Annesley C. (2017). Intent-to-treat leukemia remission by CD19 CAR T cells of defined formulation and dose in children and young adults. Blood.

[3] Sanber K., Savani B., Jain T. (2021). Graft-versus-host disease risk after chimeric antigen receptor T-cell therapy: the diametric opposition of T cells. Br J Haematol.

[4] Jain T., Sauter C.S., Shah G.L. (2019). Safety and feasibility of chimeric antigen receptor T cell therapy after allogeneic hematopoietic cell transplantation in relapsed/ refractory B cell non-Hodgkin lymphoma. Leukemia.

[5] Peñas P.F., Jones-Caballero M., Aragüés M., Fernández-Herrera J., Fraga J., García-Díez A. (2002). Sclerodermatous graft-vs-host disease: clinical and pathological study of 17 patients. Arch Dermatol.

[6] Schneider J., Kuhlmann L., Xiao Y. (2022). Healthy-like CD4^+^ regulatory and CD4^+^ conventional T-cell receptor repertoires predict protection from GVHD following donor lymphocyte infusion. Int J Mol Sci.

[7] Schmid C., Labopin M., Schaap N. (2022). Long-term results and GvHD after prophylactic and preemptive donor lymphocyte infusion after allogeneic stem cell transplantation for acute leukemia. Bone Marrow Transplant.

[8] Lutfi F., Holtzman N., Siglin J. (2021). Chimeric antigen receptor T-cell therapy after allogeneic stem cell transplant for relapsed/refractory large B-cell lymphoma. Br J Haematol.

